# Factors Influencing the Use of Health Information Exchange by Physicians—Using the National Health Insurance PharmaCloud System in Taiwan

**DOI:** 10.3390/ijerph18168415

**Published:** 2021-08-09

**Authors:** Chiou-Hwa Chuang, Yi-Fan Li, Lu-Cheng Kuo, Ming-Chin Yang, Li-Ting Kao

**Affiliations:** 1 Medical Information Management Office, National Taiwan University Hospital, Taipei 100225, Taiwan; chiouhwa@ntu.edu.tw; 2 National Defense Medical Center, School of Public Health, Taipei 114201, Taiwan; 3 Division of Clinical Chinese Medicine, National Research Institute of Chinese Medicine (NRICM), Ministry of Health and Welfare, Taipei 112304, Taiwan; yifanli@nricm.edu.tw; 4 National Taiwan University Hospital and College of Medicine, Taipei 100025, Taiwan; kuolc@ntu.edu.tw; 5 Institute of Health Policy and Management, College of Public Health, National Taiwan University, Taipei 100025, Taiwan; 6 National Defense Medical Center, Graduate Institute of Life Sciences, Taipei 114201, Taiwan; 7 Department of Pharmacy Practice, Tri-Service General Hospital, Taipei 114202, Taiwan; 8 National Defense Medical Center, School of Pharmacy, Taipei 114201, Taiwan

**Keywords:** PharmaCloud, health information exchange, electronic medical records, technology continuance theory, technology acceptance model, MediCloud system, health information systems

## Abstract

This study aimed to investigate the factors influencing physicians use of the PharmaCloud system in Taiwan through Technology Continuance Theory (TCT) and to construct a TCT-based structured questionnaire to demonstrate the attitude and behavior of physicians in the Taiwanese medical system. It focused on investigating “confirmation”, “perceived usefulness”, “perceived ease of use”, “attitude”, “satisfaction”, and “continuance intention” towards the preload-based comparison and manual search in PharmaCloud by attending physicians during their outpatient clinics. Path analysis was used to analyze the cause and effect relationship between variables. This study collected 528 valid questionnaires and the results of path analysis found that factors affecting physicians’ continued use of preload-based comparison in PharmaCloud included “perceived usefulness”, “satisfaction”, and “attitude” (all *p* < 0.001); however, factors that influenced physicians’ continued use of manual search in PharmaCloud were only “satisfaction” and “attitude” (all *p* < 0.001). Additionally, the effects of “perceived usefulness” and “perceived ease of use” on “satisfaction” could only be seen in preload-based comparison in PharmaCloud. In conclusion, when physicians’ actual use of PharmaCloud met their expectations, physicians had higher levels of confirmation and better perceived usefulness, which naturally increased their satisfaction and attitude towards PharmaCloud and positively prompted them to continue using it.

## 1. Introduction

The National Health Insurance (NHI) Administration in Taiwan constructed the PharmaCloud System in July 2013 to integrate the medical records of patients when treatments are sought at different medical institutions [1]. Initially, the NHI database compiled the medication information of each patient from clinics, hospitals, and pharmacies and then uploaded this information to the cloud system, creating a dedicated medication file for each patient. The medication used by a patient during the last three months can be queried, including medication from the hospital where the query was made, as well as from other hospitals [1,2].

The PharmaCloud System was upgraded to the NHI-MediCloud System in 2016. Since then, physicians can look at the past medical records of the patient when they provide clinical treatment or prescribe medication, divided into eleven categories: medication records in Western medicine, medication records in traditional Chinese medicine, test and examination results, detailed surgery records, dental records, drug allergy records, usage records of specific controlled drugs, usage records of specific coagulation factors, rehabilitation records, and discharge summaries. In January 2018, the system also began allowing users to query medical examination images, such as computed tomography (CT) scans and magnetic resonance imaging (MRI) images [3].

Medical institutions can use this system in two ways. The first is via batch download queries (referred to as preload-based comparison in this study). With written consent obtained from a patient at any hospital, medical institutions can download the patient’s medication records in one whole batch before the patient’s visit, based on his or her appointment number. During the visit, the physician can then make comparisons and see what other medication the patient is currently, or was previously, taking in order to prevent double dosing or adverse drug interactions. The second method is instant manual queries (referred to as manual search in this study). For patients who did not make an appointment in advance, physicians can use their personal physician IC card and the patient’s NHI card to manually access the patient’s medication records in the online cloud database. Both methods enable physicians to check for double dosing and drug interactions. As physicians can easily know what medication patients have taken, they can ask the patients whether the medication was effective or produced any adverse effects [4].

Because the willingness of a physician is a crucial determinant of the success of promoting the PharmaCloud System, it is necessary to identify the factors that influence the use of the PharmaCloud System from the perspective of the physicians, including physician attitude, perceived usefulness of the PharmaCloud System in increasing healthcare quality, and their intention to continue using the system. Therefore, the study aims to examine the factors that influence physicians’ continued use, support, and acceptance of the PharmaCloud System.

There have been studies using the Technology acceptance model (TAM) to investigate factors affecting physician’s intention to use medical records [5,6,7,8]. However, since the implementation of the PharmaCloud system by the National Health Insurance Administration (NHIA) of Taiwan in 2013, no study has been done to examine whether physicians are willing to use this system to query the medication history of patients and their willingness to continuously use it. Therefore, this study will use Technology Continuance Theory (TCT) to examine factors affecting physician’s intention to continuously use the PharmaCloud system in order to query patients’ medication history.

## 2. Materials and Methods

### 2.1. Study Design

This study aimed to identify a research model to help understand physicians’ intentions to use the PharmaCloud system continuously. When considering the PharmaCloud system as a type of medical record, we found that TAM was widely used to explain and predict physicians’ attitudes and behavior in terms of adopting electronic medical records [5,9,10,11,12]. However, TAM focuses only on the intention to adopt a new technology rather than measuring the willingness to continuously use such technology. Therefore, we chose to use Technology Continuance Theory (TCT) proposed by Liao et al. in 2009 which combines TAM, Expectation Confirmation Model (ECM) and Cognitive Model of Satisfaction Decisions (COG), to evaluate physicians’ continuance usage intention regarding the PharmaCloud system [13].

TCT can also measure satisfaction and attitudes toward a new technology [14]. By measuring the key concepts of TAM, ECM, and COG, i.e., satisfaction and attitudes, TCT can predict more effectively an individual’s intention to continuously use a technology [14]. Therefore, this study adopted TCT and used its six key concepts to identify variables and formulate our questionnaire, namely Confirmation, Perceived usefulness, Perceived ease of use, Satisfaction, Attitude, and Continuance intention.

We conducted a cross-sectional survey at six hospitals which belong to the medical healthcare system in Taiwan, including a main medical center, two regional hospitals, and three district hospitals. The study hypothesized that physicians’ perceived ease of use, usefulness, confirmation, and satisfaction of the PharmaCloud System influenced their utilization, attitude, and continuance intention, respectively. This study was approved by the Research Ethics Committee at the National Taiwan University Hospital (2017050007RINA).

### 2.2. Participants and Data Collection

The study initially enrolled 775 specialists and visiting physicians who were employed full-time in the six hospitals. A self-reported questionnaire was designed to measure variables. The survey was conducted through morning meetings at each hospital from August to November in 2017. We recovered 565 questionnaires. After eliminating 37 invalid questionnaires, we obtained 528 valid questionnaires in total, thereby producing a total response rate of 68.13%.

### 2.3. Instrument Development and Research Variables

The questionnaire comprised two parts involving the basic characteristics of the physicians and the six constructs. The question items were measured using a 5-point Likert scale indicating their degree of agreement with the statements in the items. The basic characteristics investigated in this study included the level of the hospital where they were employed, gender, age, department, years of service, the number of outpatient clinic sessions per week, and the average number of patients seen per session.

We drafted the question items based on existing literature concerning the Technology Acceptance Model (TAM) and TCT [13,15,16,17,18] with consideration given to the characteristics and practices of the target subjects. The question items were then reviewed by nine experts, revised, and then finalized. The questionnaire contained six constructs investigating physicians’ use of the preload-based comparison and manual search functions of the PharmaCloud System. Each construct comprised 18 question items. Table 1 presents the question items and their reference sources.

The research variable ‘Confirmation’ indicated that individuals get a sense of confirmation when the actual results meet expectations [19]. The definition of ‘Perceived usefulness’ is the degree to which an individual subjectively perceives that a system will enhance their work performance. ‘Perceived ease of use’ is the amount of effort that an individual subjectively perceives to be needed to use a system, as well as the perceived ease of use of the system. In this study, this means the degree to which using the PharmaCloud System will help physicians work faster, better, more easily, and more productively. ‘Attitude’ is the intensity of positive or negative emotions felt by an individual toward executing the target action and ‘Satisfaction’ indicates the attitude and opinions of an individual with regard to the performance of a product or service [20]. Furthermore, the ‘Continuance intention’ refers to whether an individual is willing to continue using a system in the future [21,22].

This study formulated the following hypotheses for both approaches, namely preload-based comparison and manual search:

**Hypothesis** **1** **(H1).** *The confirmation of physicians with regard to the PharmaCloud System influences their perceived usefulness*.

**Hypothesis** **2** **(H2).** *The perceived ease of use by physicians with regard to the PharmaCloud System influences their perceived usefulness*.

**Hypothesis** **3** **(H3).** *The perceived ease of use by physicians with regard to the PharmaCloud System influences their attitude*.

**Hypothesis** **4** **(H4).** *The perceived usefulness by physicians with regard to the PharmaCloud System influences their attitude*.

**Hypothesis** **5** **(H5).** *The perceived usefulness by physicians with regard to the PharmaCloud System influences their satisfaction*.

**Hypothesis** **6** **(H6).** *The confirmation of physicians with regard to the PharmaCloud System influences their satisfaction*.

**Hypothesis** **7** **(H7).** *The satisfaction of physicians with regard to the PharmaCloud System influences their attitude*.

**Hypothesis** **8** **(H8).** *The perceived usefulness by physicians with regard to the PharmaCloud System influences their continuance intention*.

**Hypothesis** **9** **(H9).** *The satisfaction of physicians with regard to the PharmaCloud System influences their continuance intention*.

**Hypothesis** **10** **(H10).** *The attitude of physicians with regard to the PharmaCloud System influences their continuance intention*.

### 2.4. Reliability and Validity of Questionnaire

#### 2.4.1. Reliability

To confirm the consistency of the content of the questionnaire, we used Cronbach’s α to examine the internal consistency of the six constructs. For preload-based comparison, Cronbach’s α for each of the constructs ranged from 0.858 to 0.930 (results not shown). For manual search, the values ranged from 0.818 to 0.947. Overall, the values of Cronbach’s α for all six constructs were acceptable values ranging from 0.70 to 0.95 [23,24]. However, we chose to include all the items in the following analysis because each item was formulated to measure different aspects of a construct, although the high values of alpha may cause multicollinearity. 

#### 2.4.2. Validity

Content validity was assessed using expert validity. Nine experts including scholars, the hospital executive responsible for PharmaCloud System operations, and clinical physicians were asked to rate the appropriateness of the content of the question items and the clarity of their wording, with CVI (content validity index) as the index. The CVI values of Item 18 in the first part regarding preload-based comparison and the second part regarding manual search were 0.67 and 0.55, respectively. As a result, we revised the question items as suggested by the experts. Apart from the individual CVI of one expert being 0.75, the individual CVI values of the other experts were greater than 0.8. Relevant studies recommend that the overall CVI be 0.78 or higher for a questionnaire to have good content validity [25]. The overall CVI of our questionnaire was 0.93.

Regarding convergent validity, the Kaiser-Meyer-Olkin (KMO) values of preload-based comparison and manual search were 0.96 and 0.956, respectively (both greater than 0.5). In addition, the results of the Bartlett’s test of sphericity were all significant, indicating that factor analysis could be applied to the question items in the questionnaire. The confirmatory factor analysis (CFA) results revealed that the factor loadings of the question items were all greater than 0.7, the average variance extracted (AVE) values of the six constructs were all greater than 0.5, the SMC values were all greater than 0.5, and the composite reliability (CR) values were all greater than 0.6. Thus, the constructs in this questionnaire all have good convergent validity (please see the Supplementary Materials for the results).

Tables S1 and S2 are the discriminant validity coefficient of preload-based search and manual search, respectively. The coefficients show that the square roots of average variance extracted (AVE) of a variable within a construct are greater than those between other constructs. Thus, these variables should have sufficient discriminant validity.

### 2.5. Statistical Method 

Reliability and validity analysis, descriptive statistics analysis, and path analysis were performed using SPSS 24.0 and AMOS 24.0. We used Cronbach’s alpha to test the reliability, CVI to test content validity, and CFA to measure the degree of fit of each construct. The factor loadings, CR and AVE obtained were utilized to test convergent validity; the square root of AVE was used to determine the discriminant validity between constructs. Descriptive statistics were used to analyze the frequency, percentage, mean, and standard deviation of the basic characteristics. Path analysis was employed to examine the causal relationships among the variables and to verify the appropriateness of the theoretical model. Furthermore, for path coefficient analysis, we used the bootstrapping method in Amos to take samples 1000 times and calculate the standardized path coefficient (β). Path coefficients reveal the intensity and direction of relationships between research variables. The standardized path coefficient (β) and the *p* value were used to determine the significance of variable relationships, with *p* < 0.001 indicating that the variable path relationship is significant. Then, this study employed R^2^ to gauge the effectiveness and explanatory power of the model.

## 3. Results

### 3.1. Respondents Characteristics

We received 565 questionnaires of the 775 distributed. After eliminating 37 invalid questionnaires, we obtained 528 valid questionnaires in total, thereby producing a valid response rate of 68.13%. The characteristics of respondents in this study are addressed in Table 2. A total of 46.8% and 40.5% of respondents were physicians in regional hospitals and medical centers, respectively. The mean age of the recruited physicians was about 42 years old and the mean years of service was about 11. Most physicians were male (80%).

### 3.2. Path Analysis Results

**Figure 1:**
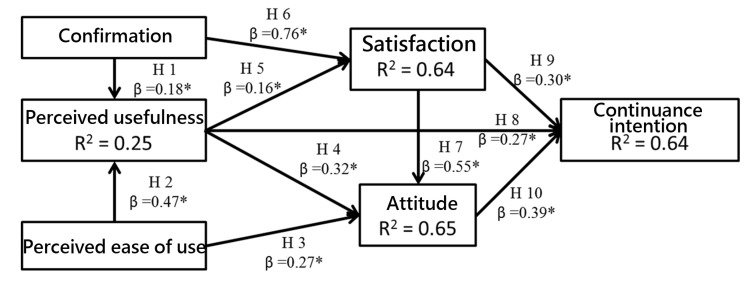
Path analysis of variables in research framework (preload-based comparison); Note: * *p* < 0.001.

**Figure 2:**
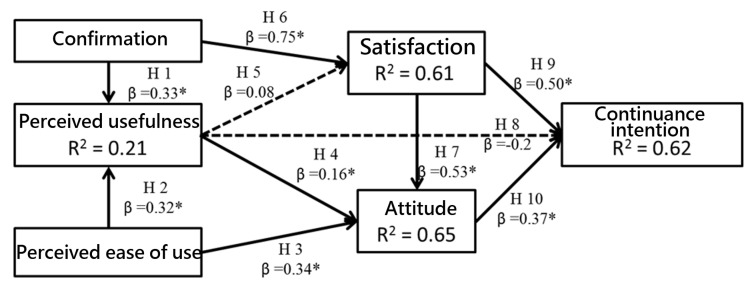
Path analysis of variables in research framework (manual search); Note: * *p* < 0.001.

The path analysis results of the research framework for preload-based comparison and manual search are displayed in Figure 1 and Figure 2, respectively. Both figures include the path coefficients, R^2^ values, and *p* values. *p* values less than 0.001 indicate significance and are presented using bold lines, meaning that the relevant hypothesis is supported. In contrast, *p* values greater than 0.001 indicate no significance and are presented using dashed lines, meaning that the relevant hypothesis is not supported. In addition, according to Table 3 and Table 4, the squared multiple correlation (SMC), AVE and critical ratio (CR) are appropriately large (SMC > 0.2; AVE > 0.5; CR> 0.7). These findings indicated that the measurement model for both preload-based comparison and manual search in the PharmaCloud System is valid.

In Figure 1 and Figure 2, except for H5 (*The perceived usefulness by physicians with regard to the PharmaCloud System influences their satisfaction*) and H8 (*The perceived usefulness by physicians with regard to the PharmaCloud System influences their continuance intention*) in manual search, all of the hypotheses were supported. We explain each of the hypothesis testing results in more detail below.

The variables that influenced the perceived usefulness of preload-based comparison and manual search in the PharmaCloud System included confirmation (preload-based comparison H1: β = 0.18, *p* < 0.001; manual search H1: β = 0.33; *p* < 0.001) and perceived ease of use (preload-based comparison H2: β = 0.47, *p* < 0.001; manual search H2: β = 0.32, *p* < 0.001). These findings demonstrate that the confirmation and perceived ease of use by physicians with regard to preload-based comparison and manual search exert a positive impact on their perceived usefulness.

The variables that influenced the attitude toward preload-based comparison and manual search in the PharmaCloud System included perceived ease of use (preload-based comparison H3: β = 0.27, *p* < 0.001; manual search H3: β = 0.34, *p* < 0.001), perceived usefulness (preload-based comparison H4: β = 0.32, *p* < 0.001; manual search H4: β = 0.16, *p* < 0.001), and satisfaction (preload-based comparison H7: β = 0.55, *p* < 0.001; manual search H7: β = 0.53, *p* < 0.001). These findings indicate that the perceived ease of use, perceived usefulness, and satisfaction of physicians with regard to preload-based comparison and manual search exert a positive impact on their attitude, and thus prompt them to adopt a better attitude toward the system.

Confirmation influenced the satisfaction with preload-based comparison and manual search in the PharmaCloud System (preload-based comparison H6: β = 0.76, *p* < 0.001; manual search H6: β = 0.75, *p* < 0.001). While perceived usefulness influenced satisfaction of preload-based comparison, its influence on satisfaction of manual search was not significant (preload-based comparison H5: β = 0.16, *p* < 0.001; manual search H5: β = 0.08, *p* = 0.005). This reveals that greater usefulness perceived by physicians in preload-based comparison increased their satisfaction with preload-based comparison; however, greater usefulness perceived by physicians in manual search did not influence their satisfaction. Furthermore, physicians displayed greater satisfaction when the use of preload-based comparison and manual search exceeded their expectations.

The variables that influenced the continuance intention with regard to preload-based comparison and manual search in the PharmaCloud System included satisfaction (preload-based comparison H9: β = 0.30, *p* < 0.001; manual search H9: β = 0.50, *p* < 0.001) and attitude (preload-based comparison H10: β = 0.39, *p* < 0.001; manual search H10: β = 0.37, *p* < 0.001). Although perceived usefulness influenced continuance intention with regard to preload-based comparison, its influence on continuance intention with regard to manual search was not significant (preload-based comparison H8: β = 0.27, *p* < 0.001; manual search H8: β = −0.20, *p* = 0.551). This indicates that greater usefulness perceived by physicians in preload-based comparison increased their continuance intention, but this was not apparent for manual search.

All variable relationships in preload-based comparison were significant, whereas the influence of perceived usefulness on satisfaction and continuance intention was not significant. Then, this study used R^2^ to estimate the effectiveness and explanatory power of the model. Aside from the perceived usefulness of preload-based comparison and manual search, all of the other variables had R^2^ coefficients greater than 0.5, which indicates that TCT has good explanatory power to effectively identify variables that affect the continuance intention of physicians using the PharmaCloud system. 

## 4. Discussion

The findings of this study revealed that preload-based comparison was easier to use than manual search and greater perceived ease of use promoted greater perceived usefulness. According to the results of SEM, we found that all the hypotheses were verified in the preload-based comparison. This means that confirmation, perceived usefulness, perceived ease of use, attitude, and satisfaction all significantly influenced continuance intention. However, in manual search, the relationships were between physicians’ perceived usefulness and satisfaction, and continuance intention (H5 & H8). This finding is consistent with those of many TAM and TCT studies [8,12,15,26] which found that attitude and satisfaction were the primary influence factors of continuance intention.

Our results revealed that the influence of perceived usefulness on continuance intention and satisfaction is only significant with preload-based comparison and is not significant with manual search, even though many studies have indicated that perceived usefulness is the primary factor that can best predict usage intention or continuance intention [5,15,16]. This result is inconsistent with that derived by Tung et al., who discovered that perceived usefulness and perceived ease of use both exert a significant and positive impact on the intention of physicians to use electronic medical record exchange [27]. The relationship between perceived ease of use and perceived usefulness has been shown to be significant in many existing studies, and their influence on each other is extremely strong, i.e., highly correlative [5,15,26]. However, Davis advocated that the relationship between perceived ease of use and perceived usefulness is not just correlation. Following in-depth investigations, some researchers have found that perceived ease of use influences usage intention indirectly via perceived usefulness [8,15,28], suggesting that perceived ease of use mediates the relationship between perceived usefulness and usage intention. Future studies should consider examining the impacts of both perceived usefulness and perceived ease of use on continuance intention. 

In a study on the attitude of medical personnel toward an electronic medical record system in a hospital in Iran, Gilani et al. identified perceived ease of use, perceived usefulness, and satisfaction as the primary influence factors on attitude [26]. In contrast to the findings of Gilani et al. and this study, Hu et al. found that perceived usefulness has an extremely significant impact on attitude with regard to a medical information system; however, perceived ease of use had no significant influence on attitude or perceived usefulness. Our findings, though, demonstrate that the ease of use perceived by physicians in the PharmaCloud System had a significant impact on perceived usefulness and attitude [29].

Ayanso et al. discovered that confirmation and perceived usefulness both exert a positive influence on satisfaction [30]. Similarly, we found that the confirmation experienced by physicians with regard to preload-based comparison and manual search had a positive impact on their satisfaction. In other words, the more that preload-based comparison and manual search exceeded the expectations of the physicians, the more satisfied they were with the system. However, with regard to perceived usefulness, we found that the positive influence of perceived usefulness on satisfaction was only present in preload-based comparison and not in manual search. Gilani et al. posited that, as physicians use electronic medical records for longer periods of time and become more experienced with them, the influence of confirmation on satisfaction becomes stronger, but the influence of perceived usefulness on satisfaction becomes weaker [26]. With regard to satisfaction, our findings were consistent with those obtained by Heselmans et al. and Nurdin et al., in that continuance intention is strongly affected by satisfaction, and satisfaction was generated from perceived usefulness and perceived ease of use. In other words, our study found that physicians who are more satisfied with electronic medical records are more likely to continue using them [31,32].

Hsieh studied the factors that influence the use of electronic medical record exchange by physicians in Taiwan and concluded that attitude, perceived usefulness, and perceived ease of use are all factors of usage intention and that these variables are impacted by self-efficacy, trust, and organization [21]. Our study found some resonance with these studies, in that the continued use of preload-based comparison and manual search in the PharmaCloud System by physicians is influenced by perceived usefulness, attitude, and satisfaction.

This study has several limitations. The first was that, although the respondents encompassed physicians employed at a medical center, regional hospitals, and district hospitals, they were all part of the same healthcare system, and we did not examine any hospitals outside of this healthcare system. Thus, the generalizability of the findings is limited and may not represent all of the physicians using the PharmaCloud System in Taiwan. The second limitation was that a cross-sectional design was adopted for this study. Although we used path analysis to present the theoretical causal relationships, long-term trends in the use of the PharmaCloud System by physicians would be better presented if longitudinal data were available.

## 5. Conclusions

This study found that when the actual usage of the PharmaCloud System was closer to the expectations of physicians, the physicians perceived greater usefulness in the system, displayed better satisfaction and attitude toward the system, and expressed a greater likelihood that they would continue using the system for a longer period of time. Accordingly, we put forward some suggestions for health authorities, medical institutions, and future research. First, besides effectiveness, the most important consideration in promoting cloud medication records is the ease of use of a system. It must not be too cumbersome in clinical use and must be of practical value in clinical diagnosis to increase the willingness of physicians to use them. Thus, user feedback and the needs of clinical practice must be taken into consideration to enhance the practicality and integration of a system. Second, the PharmaCloud System should be well-integrated with the medical information system, and physicians should be provided with a quick and easy-to-use interface to not negatively affect their clinical tasks. As physicians and medical personnel are the primary users of the PharmaCloud System, we therefore suggest that electronic medical record exchange should be designed with the needs of these users in mind.

This study verified the relationships among main influencing factors of TCT. In terms of preload-based comparison, when physicians confirm that the PharmaCloud system is up to their expectation, their perceived usefulness will be higher, and in turn satisfaction and attitude will be better. This results in a positive influence on continuance intention to use the PharmaCloud system. On the other hand, in the manual search approach, Perceived usefulness did not influence Satisfaction and Continuance intention. Therefore, the study results indicated that physicians had a more positive attitude towards the preload-based comparison.

Furthermore, the results of this study can provide government agencies or hospital authority with a useful reference when implementing a PharmaCloud system or similar. Physicians would be more willing to search and compare what medications their patients had before prescribing a new treatment if the system is a preload-based comparison. More importantly, a preload-based comparison approach will increase physicians’ continuance intention in using the system. Hospital authorities can conduct periodic surveys to understand physicians’ need for support, resources, or training, and provide this support in a timely fashion, so that physicians will be more willing to use the system and ultimately enhance the quality of care to patients.

## Figures and Tables

**Table 1:** Question items and their reference sources.

Variable	Question Item	Reference Source
1. Confirmation	(1) My experience with using preload-based comparison/manual search in PharmaCloud was better than what I expected.	[13]
	(2) The functions provided by preload-based comparison/manual search in PharmaCloud was better that what I expected.	
	(3) Overall, most of my expectations from using preload-based comparison/manual search in PharmaCloud were confirmed.	
2. Perceived usefulness	(4) Using preload-based comparison/manual search in PharmaCloud improves the accuracy of a prescription.	[15,16]
	(5) Using preload-based comparison/manual search in PharmaCloud helps avoid or mitigate repeated medication.	
	(6) Using preload-based comparison/manual search in PharmaCloud helps improve the quality of patient care.	
3. Perceived ease of use	(7) I find the information provided by preload-based comparison/manual search in PharmaCloud clear and understandable.	[15,16]
	(8) Interaction with preload-based comparison/manual search in PharmaCloud does not require a lot of my mental effort.	
	(9) I find preload-based comparison/manual search in PharmaCloud easy to use.	
4. Attitude	(10) Using preload-based comparison/manual search in PharmaCloud to avoid or mitigate repeated medication would be a good idea.	[13]
	(11) I like using preload-based comparison/manual search in PharmaCloud during consultations.	
	(12) Using preload-based comparison/manual search in PharmaCloud has been a pleasant experience.	
5. Satisfaction	(13) My overall experience of the function of preload-based comparison/manual search in PharmaCloud was very satisfying.	[13,18]
	(14) My overall experience of the content of preload-based comparison/manual search in PharmaCloud was very satisfying.	
	(15) My overall experience of preload-based comparison/manual search in PharmaCloud use was very satisfying.	
6. Continuance intention	(16) I am very willing to continue using preload-based comparison/manual search in PharmaCloud.	[13,17]
	(17) I intend to continue using preload-based comparison/manual search in PharmaCloud than discontinue its use.	
	(18) My intentions are to continue using preload-based comparison/manual search in PharmaCloud than use any alternative means.	

**Table 2:** Characteristics of respondents.

Respondents Characteristics	Number of People ^a^	%
Gender		
Male	415	80.0
Female	104	20.0
Age		
<31 years old	22	4.3
31–40 years old	244	47.6
41–50 years old	171	33.3
>50 years old	76	14.4
Years of service		
<6 years	119	23.5
6–10 years	170	33.5
11–20 years	162	32.0
>20 years	56	11.0
Department		
Department of internal medicine	161	30.5
Department of surgery	82	15.5
Other internal medicine field	132	25.0
Other surgery field	132	25.0
Number of outpatient clinic sessions per week	511	100.0
1 time	51	10.0
2 times	223	43.6
3 times	158	30.9
>3 times	79	15.5
Average number of patients seen per session		
<21 patients	134	26.1
21–30 patients	140	27.2
31–40 patients	120	23.3
41–50 patients	65	12.6
>50	55	10.7
Hospital level		
Medical center	214	40.5
Regional hospital	247	46.8
District hospital	67	12.7

Note: ^a^ The *n* values do not add up to the total recruited number because of the missing data.

**Table 3:** Confirmatory factor analysis results for preload-based comparison.

Item	Factor Loading	Squared Multiple Correlation (SMC)	Average Variance Extracted (AVE)	Composite Reliability (CR)
Perceived usefulness				
(1) Using manual search in PharmaCloud improves the accuracy of a prescription.	0.88	0.729	0.802	0.892
(2) Using manual search in PharmaCloud helps avoid or mitigate repeated medication.	0.83	0.849		
(3) Using manual search in PharmaCloud helps improve the quality of patient care.	0.86	0.828		
Perceived ease of use				
(4) I find the information provided by the manual search in PharmaCloud clear and understandable.	0.76	0.889	0.880	0.888
(5) Interaction with the manual search in PharmaCloud does not require a lot of my mental effort.	0.91	0.886		
(6) I find the manual search in PharmaCloud easy to use.	0.88	0.864		
Confirmation				
(7) My experience with using manual search in PharmaCloud was better than what I expected.	0.92	0.810	0.690	0.950
(8) The functions provide by manual search in PharmaCloud was better that what I expected.	0.95	0.718		
(9) Overall, most of my expectations from using manual search in PharmaCloud were confirmed.	0.92	0.541		
Attitude				
(10) Using preload-based comparison in PharmaCloud to avoid or mitigate repeated medication would be a good idea.	0.74	0.847	0.868	0.871
(11) I like using preload-based comparison in PharmaCloud during consultations.	0.85	0.908		
(12) Using preload-based comparison in PharmaCloud has been a pleasant experience.	0.90	0.849		
Satisfaction				
(13) My overall experience of the function of the preload-based comparison in PharmaCloud was very satisfying.	0.93	0.770	0.724	0.956
(14) My overall experience of the content of the preload-based comparison in PharmaCloud was very satisfying.	0.94	0.824		
(15) My overall experience of the preload-based comparison in PharmaCloud use was very satisfying.	0.94	0.578		
Continuance intention				
(16) I am very willing to continue using preload-based comparison in PharmaCloud.	0.91	0.746	0.736	0.922
(17) I intend to continue using preload-based comparison in PharmaCloud than discontinue its use.	0.92	0.683		
(18) My intentions are to continue using preload-based comparison in PharmaCloud than use any alternative means.	0.85	0.779		

**Table 4:** Confirmatory factor analysis results for manual search.

Item	Factor Loading	Squared Multiple Correlation (SMC)	Average Variance Extracted (AVE)	Composite Reliability (CR)
Perceived usefulness				
(1) Using manual search in PharmaCloud improves the accuracy of a prescription.	0.95	0.615	0.796	0.943
(2) Using manual search in PharmaCloud helps avoid or mitigate repeated medication.	0.92	0.860		
(3) Using manual search in PharmaCloud helps improve the quality of patient care.	0.89	0.912		
Perceived ease of use				
(4) I find the information provided by the manual search in PharmaCloud clear and understandable.	0.70	0.876	0.881	0.882
(5) Interaction with the manual search in PharmaCloud does not require a lot of my mental effort.	0.88	0.876		
(6) I find the manual search in PharmaCloud easy to use.	0.94	0.891		
Confirmation				
(7) My experience with using manual search in PharmaCloud was better than what I expected.	0.92	0.890	0.781	0.943
(8) The functions provide by manual search in PharmaCloud was better that what I expected.	0.93	0.790		
(9) Overall, most of my expectations from using manual search in PharmaCloud were confirmed.	0.91	0.664		
Attitude				
(10) Using preload-based comparison in PharmaCloud to avoid or mitigate repeated medication would be a good idea.	0.81	0.830	0.850	0.913
(11) I like using preload-based comparison in PharmaCloud during consultations.	0.89	0.867		
(12) Using preload-based comparison in PharmaCloud has been a pleasant experience.	0.94	0.852		
Satisfaction				
(13) My overall experience of the function of the preload-based comparison in PharmaCloud was very satisfying.	0.94	0.882	0.715	0.958
(14) My overall experience of the content of the preload-based comparison in PharmaCloud was very satisfying.	0.94	0.773		
(15) My overall experience of the preload-based comparison in PharmaCloud use was very satisfying.	0.94	0.490		
Continuance intention				
(16) I am very willing to continue using preload-based comparison in PharmaCloud.	0.95	0.795	0.852	0.919
(17) I intend to continue using preload-based comparison in PharmaCloud than discontinue its use.	0.93	0.849		
(18) My intentions are to continue using preload-based comparison in PharmaCloud than use any alternative means.	0.78	0.911		

## Supplementary Materials

The following are available online at https://www.mdpi.com/article/10.3390/ijerph18168415/s1, Table S1: Discriminant Validity Coefficients of Preload-based search, Table S2: Discriminant validity coefficients of Manual search.
