# Supplementary material for: Factors Influencing the Use of Health Information Exchange by Physicians—Using the National Health Insurance PharmaCloud System in Taiwan
Source: Int J Environ Res Public Health. 2021 Aug 9;18(16):8415. doi: 10.3390/ijerph18168415 (PMC8393866; doi:10.3390/ijerph18168415)
Supplement: Supplementary file 1 [file ijerph-18-08415-s001.zip › ijerph-1280287-supplementary.pdf]

Supplementary Materials

Supplementary Table S1. Discriminant Validity Coefficients of Preload-based search

	Perceived usefulness	Perceived ease of use	Confirmation	Attitude	Satisfaction	Continuance intention
Perceived usefulness	**0.896**					
Perceived ease of use	0.617	**0.938**				
Confirmation	0.575	0.870	**0.831**			
Attitude	0.732	0.878	0.900	**0.932**		
Satisfaction	0.593	0.832	0.865	0.933	**0.851**	
Continuance intention	0.757	0.780	0.795	0.917	0.825	**0.858**

Note: Values in bold are the square root of average variant extract (AVE) of that construct. Other values are the correlation coefficients between two constructs.

Supplementary Table S2. Discriminant validity coefficients of Manual search

	Perceived usefulness	Perceived ease of use	Confirmation	Attitude	Satisfaction	Continuance intention
Perceived usefulness	**0.892**					
Perceived ease of use	0.517	**0.939**				
Confirmation	0.588	0.905	**0.884**			
Attitude	0.564	0.889	0.878	**0.923**		
Satisfaction	0.507	0.821	0.850	0.936	**0.846**	
Continuance intention	0.492	0.758	0.755	0.887	0.855	**0.923**

Note: Values in bold are the square root of average variant extract (AVE) of that construct. Other values are the correlation coefficients between two constructs.
